# Robustness of differential gene expression analysis of RNA-seq

**DOI:** 10.1016/j.csbj.2021.05.040

**Published:** 2021-05-26

**Authors:** A. Stupnikov, C.E. McInerney, K.I. Savage, S.A. McIntosh, F. Emmert-Streib, R. Kennedy, M. Salto-Tellez, K.M. Prise, D.G. McArt

**Affiliations:** a Department of Biological and Medical Physics, Moscow Institute of Physics and Technology, Dolgoprudny, Russian Federation; b Patrick G. Johnson Centre for Cancer Research, Queen’s University, Belfast, Northern Ireland, UK; c Predictive Society and Data Analytics Lab, Faculty of Information Technology and Communication Sciences, Tampere University, Tampere, Finland

**Keywords:** RNA-seq, Precision medicine, Standardisation, Diagnostics, Differential gene expression analysis, Differential gene expression models

## Abstract

RNA-sequencing (RNA-seq) is a relatively new technology that lacks standardisation. RNA-seq can be used for Differential Gene Expression (DGE) analysis, however, no consensus exists as to which methodology ensures robust and reproducible results. Indeed, it is broadly acknowledged that DGE methods provide disparate results. Despite obstacles, RNA-seq assays are in advanced development for clinical use but further optimisation will be needed. Herein, five DGE models (DESeq2, voom + limma, edgeR, EBSeq, NOISeq) for gene-level detection were investigated for robustness to sequencing alterations using a controlled analysis of fixed count matrices. Two breast cancer datasets were analysed with full and reduced sample sizes. DGE model robustness was compared between filtering regimes and for different expression levels (high, low) using unbiased metrics. Test sensitivity estimated as relative False Discovery Rate (FDR), concordance between model outputs and comparisons of a ’population’ of slopes of relative FDRs across different library sizes, generated using linear regressions, were examined. Patterns of relative DGE model robustness proved dataset-agnostic and reliable for drawing conclusions when sample sizes were sufficiently large. Overall, the non-parametric method NOISeq was the most robust followed by edgeR, voom, EBSeq and DESeq2. Our rigorous appraisal provides information for method selection for molecular diagnostics. Metrics may prove useful towards improving the standardisation of RNA-seq for precision medicine.

## Graphical abstract

**Figure:**
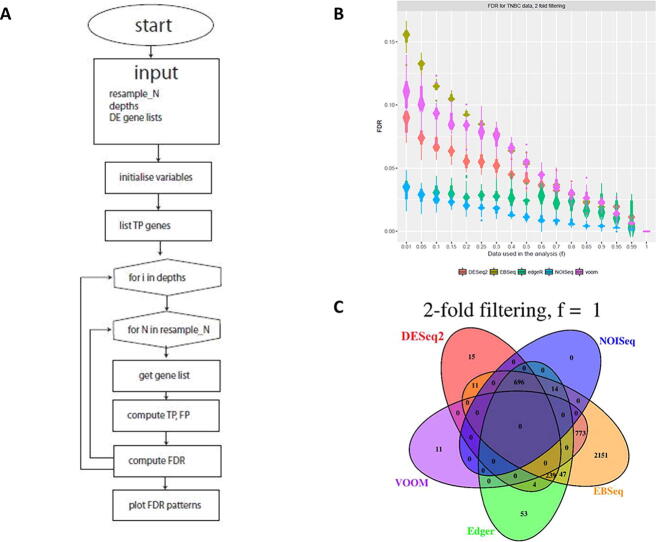

## 1 Introduction

RNA-sequencing (RNA-seq) is a high-throughput sequencing (HTS) method that measures cDNA transcripts. Transcripts are mapped to a gene/isoform and their abundance should correlate with expression. RNA-seq is a relatively new technology that has been quickly adopted into clinical research [1]. Despite this, HTS are not routinely implemented in molecular diagnostics for patient diagnosis, monitoring and management [2]. A lack of methodological standardisation and validation has previously prevented HTS adoption into the clinic [3], [4], [5]. Another major obstacle has been the complex data structure of HTS outputs (e.g. patient transcriptional profiles from RNA-seq) [5]. Regardless, RNA-seq assays are now in advanced development for precision medicine, but further optimisation is urgently needed.

RNA-seq is most often analysed to investigate expression levels of genes/transcripts between two or more conditions (i.e. contrast groups) in a Differential Gene Expression (DGE) analysis. In cancer research, DGE has been essential in assessing biological function, pathogenesis and biomarker discovery [6], [7]. To date, standardisation of RNA-seq has been problematic because results differ depending on experimental parameters used for data collection, such as HTS platform, sample loading, multiplexing and laboratory. The US FDA Sequencing Quality Control project (SEQC/MAQC-III) assessed RNA-seq performance and found that filtering DGE results improved inter-site and between-platform reproducibility [8]. Standardisation of RNA-seq for DGE is further complicated by the number and variety of analytical procedures available. DGE tools include voom + limma [9], [10], edgeR [11], Cuffdiff2 [12], EBSeq [13], SAMseq [14], Bayseq [15], NOISeq [16], rSeqNP [17], DESeq2 [18] and Sleuth [19] etc.

DGE pipelines analyse RNA-seq data with a series of steps. Initially, raw reads are aligned to a reference using popular aligners such as BWA [20], Bowtie2 [21] or STAR [22]. Aligned reads are assigned to genes from a given genome/transcriptome annotation and summarised using tools such as Cufflinks [23], HTSeq [24] or featureCounts [25]. Next, count data is normalised to enable comparisons of gene expression between samples. Different normalisation methods are available to correct for technical biases associated with gene length, library size, sequencing batches or other protocol-specific artefacts (see Methods). Normalised count data is then analysed using either a statistical model or machine learning (ML) to identify differentially expressed genes (DEGs). Models that apply parametric methods assume count distributions follow a particular distribution such as Negative binomial (NB), Log-Normal or empirical Bayes. Non-parametric methods and ML do not rely on such assumptions. DEGs are identified from results using thresholds for expression changes and/or *P*-value or posterior probability results of the test statistics.

Results of a DGE analysis are heavily influenced by the statistical model [26], [27], [28], [29]. Despite this, there is still no consensus as to which DGE methodology provides reproducibility and whether gene expression strength matters. This information is required for validation of DGE-based applications, such as molecular diagnostics. Comparing DGE pipeline robustness has been hampered by a lack of ‘Gold Standard’ datasets with known expression patterns. Such datasets are required for estimating False Discovery Rates (FDR) to assess a DGE pipeline’s performance. Instead, studies have utilised highly purified reference RNAs samples, cell lines or synthetic reads derived *in silico*. These datasets can exhibit extreme differences in gene expression between samples, hence they are unrepresentative of ‘real’ samples. Moreover, they lack the inter-sample variability in sequencing depth and quality, typically found in clinical samples. Comparing DGE pipelines has also been hampered by a lack of unbiased quantitative criteria. Studies have considered that models returning the most DEGs were best because they produced the most information [30]. This approach fails to consider that some results will be False Positives (FP) and provides no information on FDR. Another approach ranked a DGE method’s performance based on concordance of its outputs with other pipelines, examined using Venn diagrams, concordance metrics and/or hierarchical clustering dendrograms [27], [31]. This approach also does not consider FDR and software tools may cluster results only due to their model assumptions. Alternatively, simulated RNA-seq data has been used to evaluate a DGE method’s performance. Simulated datasets are advantageous for analyses because specific transcripts can be set to be differentially expressed, thereby allowing FDR estimation [31], [32]. Tools for RNA-seq simulation, such as *polyester*, assume that the number of reads for each transcript follow NB [33], the proposed distribution for RNA-seq counts [11], [34]. However, as ‘real data’ may not exactly follow NB, comparing DGE models utilising simulated datasets may give different results depending on data structure. For example, in a study to compare DGE method stability, ranks differed between the real and datasets simulated using a mixed distribution [29].

DGE method’s performance has also been compared by validating the expression of true positive results using real-time quantitative PCR (RT-qPCR) [26], [32]. This approach allows FDR to be estimated, but is limited in its utility because validation of all genes would be laborious and expensive. For RNA-seq, sufficient sequencing quality and depth has been shown to be required for DGE test recall and sensitivity [26], [30], [35]. Studies examining these parameters have not analysed clinically relevant datasets, therefore they are unable to provide a real-world test of a DGE pipeline’s performance. Lastly, computational efficiency has also been examined to compare DGE methods’ performance [31] but these metrics provide no information on the quality of a DGE model outputs.

It is essential that HTS including RNA-seq have sufficient detection power and can control FDR under variable conditions. Improving the reproducibility of HTS is necessary for the standardisation of molecular diagnostics, as well as improving the output from RNA-seq based downstream applications that require accurate gene signatures [36]. Herein, five DGE pipelines for gene-level analysis were investigated for robustness. Two clinically relevant breast cancer datasets were analysed using fixed count matrices. Results were compared with differing filtering regimes, sample sizes (full vs subset) and for genes of different expression strength using unbiased quantitative metrics. Test sensitivity estimated as relative FDR and concordance between model outputs were compared. Comparisons of a ’population’ of slopes of relative FDRs across different library sizes were also examined.

## 2 Material and methods

### 2.1 DGE pipeline normalisation methods and statistical models

Five software widely-used to determine DGE from RNA-seq data were investigated, DESeq2 v1.10.0 [18], voom + limma v3.26.0 [9], [10], edgeR 3.10.5 [11], EBSeq v1.10.0 [13] and NOISeq v2.16.0 [16]. Software differed in their normalization methods and statistical assumptions for modelling count distributions but each measured DGE at the gene-level. Normalisation is necessary because samples differ in their total numbers of sequenced reads due to technical biases. Normalisation methods to correct for larger genes having higher read counts include Transcripts per million (TPM) and Reads/Fragments Per Kilo-base per Million mapped reads (RPKM/FPKM) [37]. EBSeq applies median or quantile normalisation and NOISeq applies RPKM, TMM or upper quartile normalisation to read count data. Herein, median and upper quartile normalisation were implemented for EBSeq and NOISeq, respectively. Normalisation methods to correct for library size estimate scaling factors (based on the total number of mapped reads) and apply these globally to normalise gene expression across samples; methods include Relative Log Expression (RLE) from DESeq [34] and Trimmed Mean of M-values (TMM) [38]. edgeR applies TMM and DESeq2 applies DESeq size factors to normalise data [11], [18]. voom + limma (further referred to as ’voom’) uses an abundance gene-based method for normalization [9]. Voom determines the relationship trend of fitted log-counts per million to predict the variance of each observation and estimates a precision weight for normalisation, while taking library sizes into account. Herein, voom was applied using quantile normalisation. For each DGE pipeline, the normalisation method applied was the procedure recommended by the user’s manual.

The DGE software tested included parametric and non-parametric statistical models. edgeR and DESeq2 are parametric approaches that model count distributions using NB, but differ in their estimation of dispersion factors for characterising the mean-variance relationship (see Discussion). edgeR was implemented with the Exact test. EBSeq implements an empirical Bayesian approach for identifying DEGs that also assumes that counts are distributed according to NB [13]. Voom also adopts a parametric approach for DGE analysis; precision weights are incorporated into the Log-Normal linear model of count distributions and then an empirical Bayes statistical procedure is applied by limma [9], [10]. NOISeq adopts a non-parametric approach for DGE analysis that creates a reference distribution of the data noise by comparing the number of reads of each gene in samples in the same condition [16]. Count numbers between two conditions are then assessed against the reference distribution to determine whether they represent true differential expression or are likely to be noise.

### 2.2 RNA-seq data and TNBC and ER+ contrasts for DGE analysis

Published RNA-seq datasets were downloaded from the NCBI Gene Expression Omnibus (GEO Accession: GSE58135) [39]. Reads had been sequenced on an Illumina HiSeq 2000 using a 50 bp paired-end strategy and had ~ 50 million reads per library. Data was assembled into two independent contrasts: i) Triple Negative Breast Cancer (TNBC) primary tumours (n = 42) and their matched uninvolved breast tissue (n = 21) and ii) Estrogen Receptor Positive (ER+) Breast Cancer primary tumours (n = 42) and their matched uninvolved breast tissue (n = 30; see Table S1). TNBC and ER+ contrasts were analysed separately to determine whether observed trends were universal or dataset-dependent.

### 2.3 RNA-seq alignment and subsampling of mapped reads into count vectors

All preliminary analytical steps and parameters, such as aligner, reference genome, gene annotation and summarising approach were consistently applied in analyses. Reads were aligned using Bowtie2 [21], allowing one mismatch against the human genome version hg38 [40]. Whilst Bowtie2 is not a splice-aware aligner, reads located in splice-affected regions do not impact on transcript abundance quantification [41]. Aligned pairs of reads were mapped to genes from the *Homo sapiens* GRCh38.81 Ensembl annotation [42] using samExploreR [43]. Mapped reads were subsampled to simulate cDNA libraries with lower sequencing depth. Seventeen different fractions (*f* = 1, 0.99, 0.95, 0.9, 0.85, 0.8, 0.7, 0.6, 0.5, 0.4, 0.3, 0.25, 0.2, 0.15, 0.1, 0.05, 0.01) were randomly extracted from the Sequence Alignment / Map file with 25 iterations using samExploreR [43]. For each sample, a diverse range of library sizes, ranging from no sub-sampling and ~ 50 M reads (*f* = 1) to only 1% of reads (*f* = 0.01) were simulated (n = 425). Reads were assigned to genes and summarised into count vectors for each dataset using featureCounts 1.4.6.p5 [25]. Count matrices that provided the numerical data to be analysed by each of the DGE models were fixed, thereby facilitating a controlled analysis.

### 2.4 Count-based DGE and gene ontology analysis

Count matrices for TNBC and ER+ were analysed in a DGE analysis using each pipeline. The significance threshold (*P*-value < 0.05) applied to identify DEGs was adjusted using the Benjamini-Hochberg method to correct for multiple hypothesis testing. Gene lists of significant DEGs for each contrast, simulation (each value *f*) and iteration (each value *R* = 1,…,25) were compiled. For EBSeq, results are provided as posterior probabilities including FDRs; DEGs were determined using the threshold FDR = 0.05. Gene lists of two-fold filtered significant DEGs with Log_2_ fold change gene expression ratios greater than two (i.e. |Log_2_FC|>2) were also compiled. A functional enrichment analysis was carried out for two-fold filtered DEGs to identify their associated gene ontology (GO) terms using GOseq v1.24.0 [44].

### 2.5 Robustness, reproducibility and concordance of DEG pipeline outputs

DGE pipelines were compared for their predictions and performance for unfiltered (no-fold) and two-fold filtered DEGs, GO terms and FDRs. Results from full versus subsampled datasets facilitated the estimation of test sensitivity estimated as False Discovery Rate (FDR). FDR measures the proportion of positives that are correctly identified i.e. ‘True’ Positives (TP). In this study, results obtained for the full dataset (*f* = 1.0) are TP, while results obtained for the subsampled datasets not found by the full dataset are considered FP. FDR was computed as FP divided by the sum of FP and TP (i.e. FP/FP + TP). Herein, this FDR measured the relative false discovery rate assuming the full dataset analysed by a given method is “True”. As this measure is not FDR in the traditional sense, the measure is referred to as relative FDR throughout the text.

Results were compared using notched geometric boxplots plotted with ggplot2 [45]. Visualisation of the confidence intervals around the mean permitted assessment of the reproducibility of DGE model outputs between simulation iterations for different library sizes. Concordance between DEG pipeline outputs (DEGs, GO terms) was assessed for TNBC and ER+. Overlap in the identity of no-fold filtered and two-fold filtered DEGs and GO terms was examined using VennDiagram [46]. In addition, the concordance of two-fold filtered DEGs for low vs highly expressed genes were also compared between software. Initially genes were categorised as having low or high gene expression and compiled as a list for the TNBC and ER+ datasets separately. Using the normalised expression matrix for the control samples for each contrast (i.e. Uninvolved Breast Tissue Adjacent), mean values of the expression of genes across samples in the control samples were estimated. Using the mean expression for every gene, the median expression was then estimated. Genes were then split into low and highly expressed categories based on whether their mean expression was below or above the median threshold cut-off. Results of the differential gene expression analysis for each software were compared to the previously defined gene lists for low and high expression genes for each contrast and concordance between software for DEGs was examined using Venn diagrams.

### 2.6 Comparing sample size dependence of DGE pipeline reproducibility

DGE pipeline reproducibility was tested for sample-size dependence. Subsets of TNBC and ER+ were created by randomly selecting ten samples per contrast group (5 + 5). Subsets were analysed for DGE as previously outlined and results compared to those obtained for the full data. Performance of DGE models for larger versus smaller subsets was compared using the slopes of the regression lines of relative FDRs. This quantitative measure incorporated information on the variability of test sensitivity (i.e. FDR) with library size. To enable relative FDR estimation from results, it was necessary initially to generate a 'population' of comparative datasets for TNBC and ER+. Ten large datasets were generated by randomly removing one sample from the original sample groupings ten times. Similarly, ten subsets were generated afresh by randomly removing one sample from the original subset (5 + 4) ten times. Datasets were then subsampled (*f* = 0.8, 0.85, 0.9, 0.95, 0.99), analysed for DGE as previously outlined and results for two-fold filtered DEGs and relative FDRs estimated. A linear regression was then fitted to the relative FDR results for decreasing library sizes for each comparison and the slope of the regression line of relative FDRs estimated. For each DGE model, the slopes of 11 regression lines (ten generated datasets plus the initial full dataset) were estimated for large and subsets of each contrast. Slopes had negative values due to the inverse relationship between library size and relative FDR, such that as library sizes decrease, relative FDR increases. Hence, slopes with values close to zero would be indicative of a robust DGE method that undergoes minimal information loss following decreasing library size. By contrast, slopes with large negative values would be indicative of a DGE method that is impacted by large information loss following decreasing library size. A Friedman test was used to statistically compare the slopes of the regression lines of relative FDRs across differing library sizes (*f* = 0.8, 0.85, 0.9, 0.95, 0.99) between the population of comparative datasets and DGE models. The Friedman test is a non-parametric test analogous to two-way ANOVA, which tested the null hypothesis that mean ranks between groups were equal. The Friedman test was implemented using the R package PMCMR [47]. For significant Friedman tests, post-hoc analyses were carried out to calculate pairwise comparisons of mean rank sums using Conover and Nemenyi tests. These tests are similar but differ in the distributions they compare their test statistics to (Student's-*t* vs upper quantiles of the studentized range distribution). All computations were performed on a high performance compute cluster using R 3.2.2. Scripts utilised in this paper are hosted at https://github.com/alexstu/DGEDepth.

## 3 Results

### 3.1 Robustness and reproducibility of DGE pipelines for DEG outputs

**Fig. 1:**
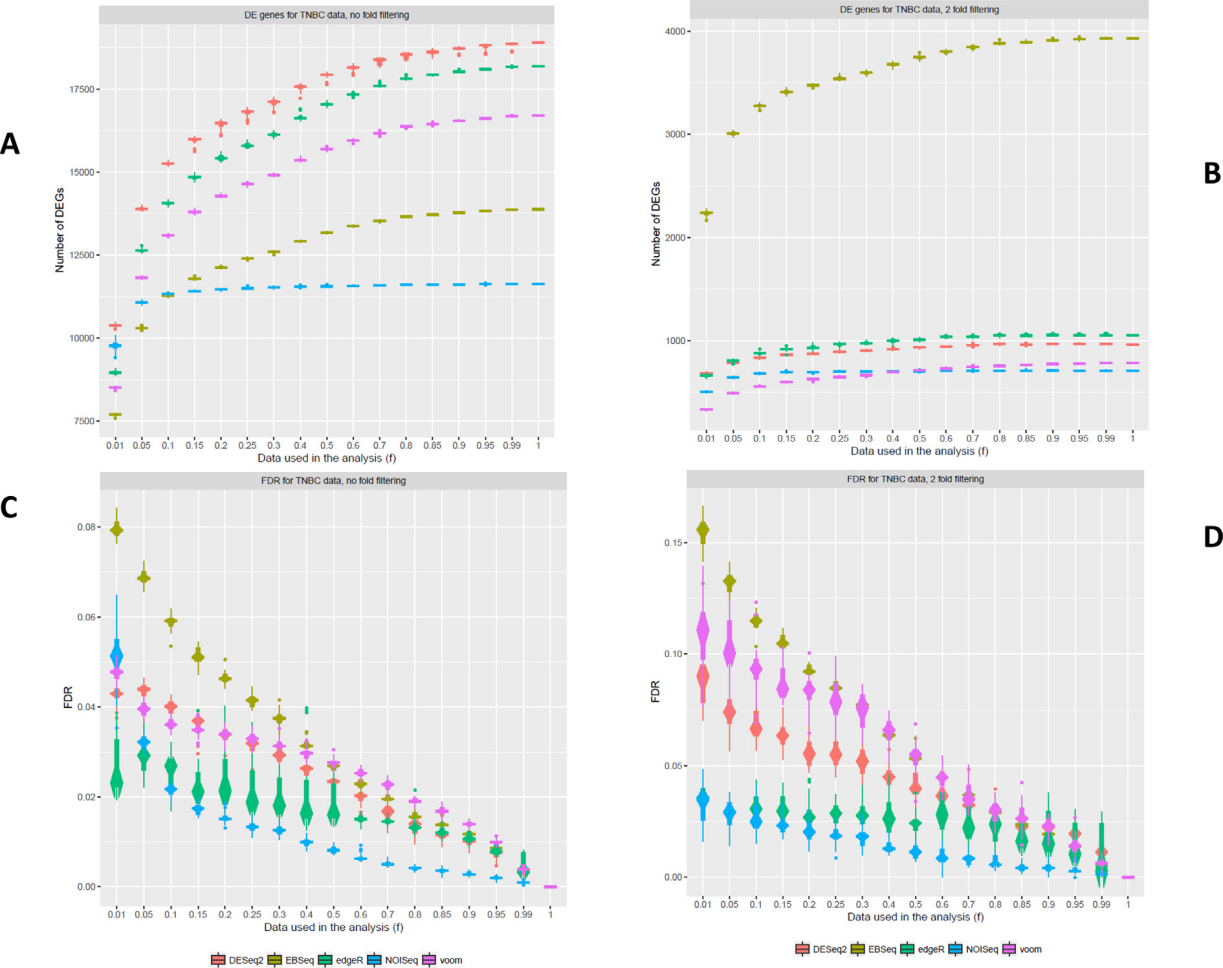
Number of DEGs detected from the TNBC dataset with differing filtering regimes – A comparison of the effect of decreased cDNA library sequencing depth on the number of DEGs detected after no-fold or two-fold filtering (a, b) from the TNBC dataset using DESeq2, edgeR, voom + limma, EBSeq and NOISeq and their associated relative FDRs (c, d).

**Fig. 2:**
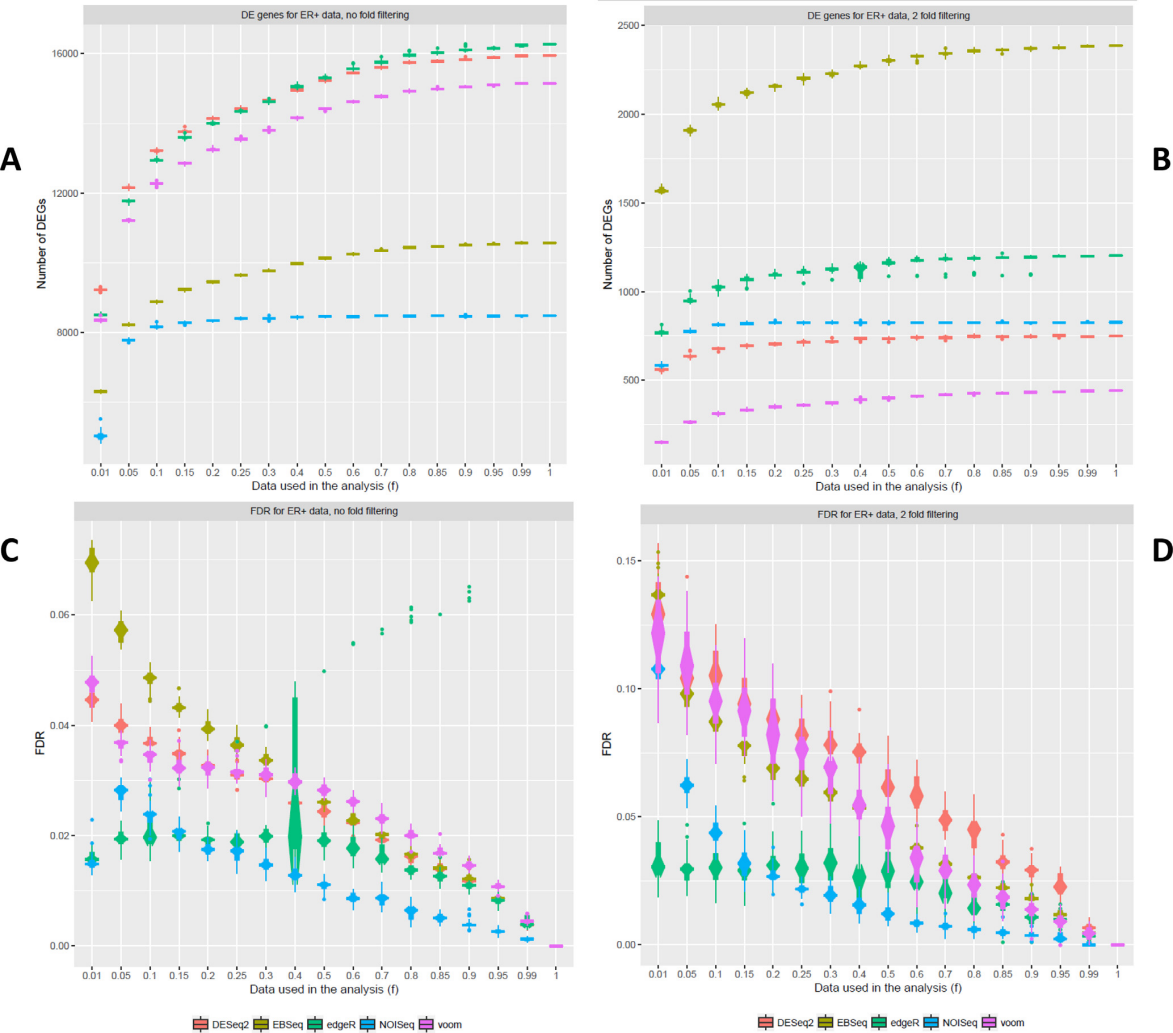
Number of DEGs detected from the ER+ dataset with differing filtering regimes – A comparison of the effect of decreased cDNA library sequencing depth on the number of DEGs detected after no-fold or two-fold filtering (a, b) from the ER+ dataset using DESeq2, edgeR, voom + limma, EBSeq and NOISeq and their associated relative FDRs (c, d).

Total number of DEGs detected differed between DGE models for both filtering regimes and data contrasts (Fig. 1, Fig. 2). The order of the DGE models that detected the greatest to the fewest DEGs also differed between filtering regimes and contrasts. For example, for TNBC with no-fold filtering and the full dataset analysed (*f* = 1), the most DEGs were detected by DESeq2 (~18,500) followed by edgeR, voom, EBSeq and NOIseq (~11,500; Fig. 1a). For TNBC after two-fold filtering and the full dataset analysed, the greatest number of DEGs was detected by EBSeq (~4,000) followed by edgeR, DESeq2, voom and NOIseq (~650; Fig. 1b). Similarly, the order of DGE models that detected the greatest to the fewest DEGs differed between filtering regimes for ER+ (Fig. 2a, b).

Patterns of test sensitivity of DGE models for no-fold and two-fold filtering were similar for both TNBC and ER+ (Figs. 1c, d; Fig. 2c, d). Across all DGE models and subsampling analyses (*f* = 1 – 0.01), observed relative FDRs were in similar ranges in both filtering regime comparisons, for TNBC (0 – ~0.08; 0 – ~0.16) and ER+ (0 – ~0.07; 0 – ~0.15), respectively. Test sensitivity was greater for no-fold filtering compared to two-fold filtering results in each contrast. Larger confidence intervals around relative FDRs were observed for two-fold filtering results, indicating greater instability for DGE model outputs. NOISeq appears to be the most stable method with consistently low relative FDR values for decreasing library sizes with both filtering regimes (Fig. 1, Fig. 2). edgeR also performed well for test sensitivity when library sizes decreased. Compared to edgeR and NOISeq, DESeq2, EBSeq and voom had relatively larger relative FDRs and confidence intervals. Thus, when library sizes decreased, test sensitivity decreased at the fastest rates for DESeq2, EBSeq and voom and their outputs were less stable compared to edgeR and NOISeq. Results were mirrored between the two independent TNBC and ER+ datasets.

### 3.2 Concordance of DGE model outputs for no-fold and two-fold filtered DEGs

**Fig. 3:**
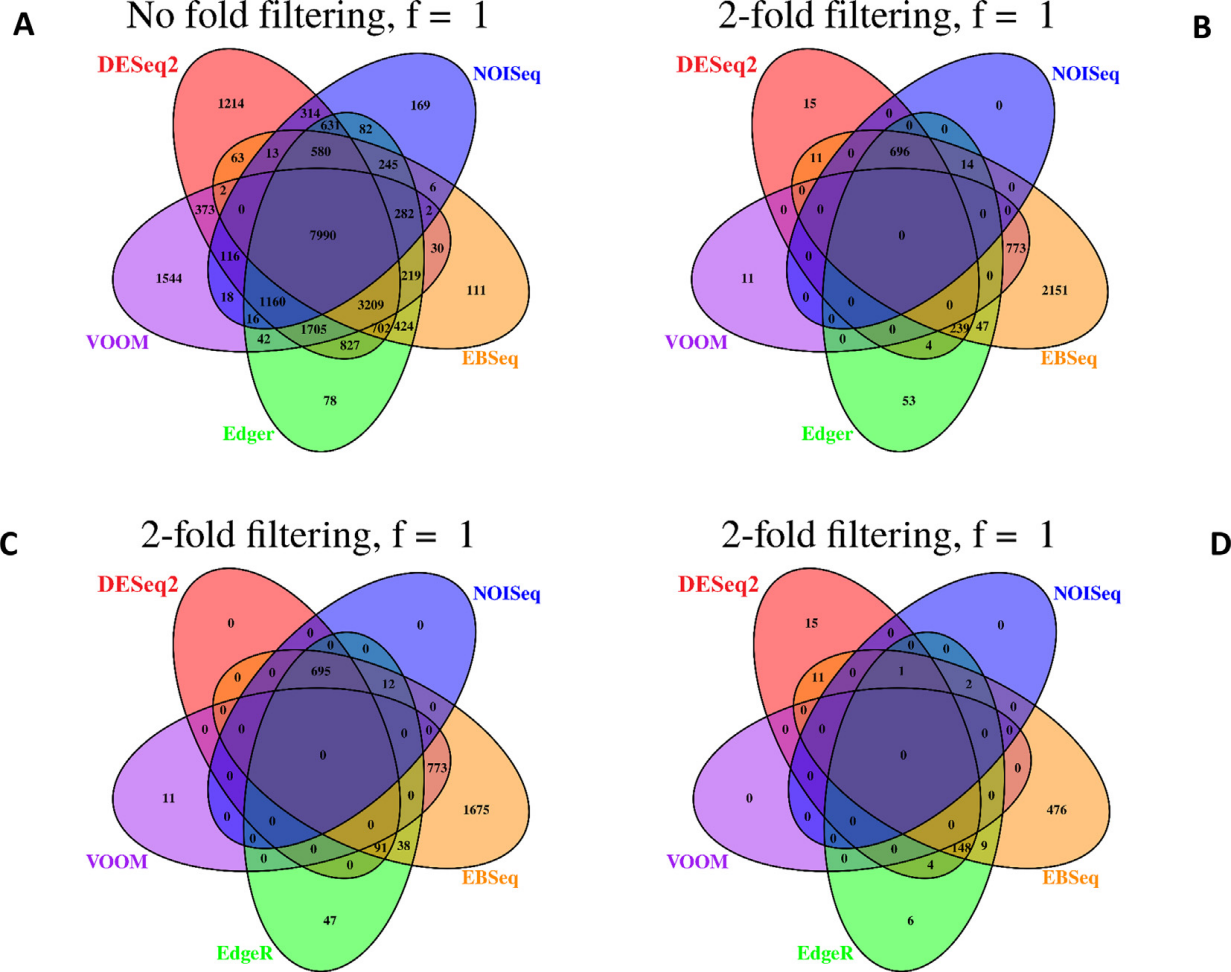
a–d. Overlap in DEGs detected from the TNBC dataset using DESeq2, edgeR, voom + limma, EBSeq and NOISeq. Results display A) no fold filtered DEGs, B) two fold filtered DEGs, C) two fold filtered DEGs with high expression, D) two fold filtered DEGs with low expression.

**Fig. 4:**
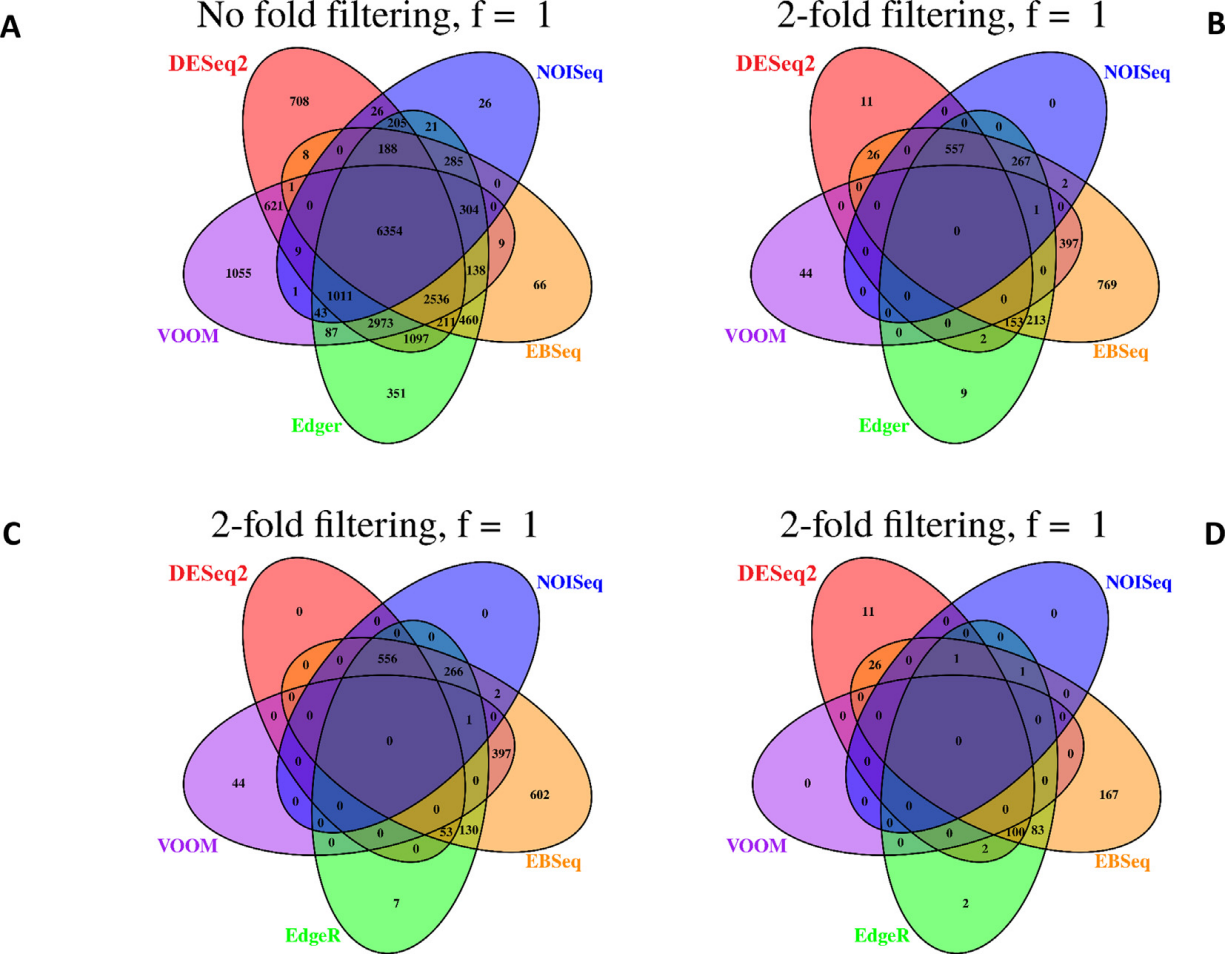
a–d. Overlap in DEGs detected from the ER+ dataset using DESeq2, edgeR, voom + limma, EBSeq and NOISeq. Results display A) no-fold filtered DEGs, B) two-fold filtered DEGs, C) two-fold filtered DEGs with high expression, D) two-fold filtered DEGs with low expression.

Comparison of no-fold filtered DEGs detected from TNBC and ER+ revealed concordance between all models (Figs. 3a and 4a), however, following two-fold filtering there was no concordance between DEGs (Figs. 3b and 4b). When fewer DGE models were considered, concordance between two-fold filtered outputs was observed, however, trends differed slightly between datasets (Figs. 3b and 4b). For TNBC, EBseq and voom had the highest number of overlapping DEGs (n = 773) followed by DESeq2, edgeR, EBSeq and NOISeq (n = 696). For ER+, the opposite was the case with DESeq2, edgeR, EBSeq and NOISeq having the highest number of overlapping DEGs (n = 557) followed by EBseq and voom (n = 397).

Trends in the number of unique DEGs detected differed considerably between DGE models and between filtering regimes (Fig. 3, Fig. 4). For two-fold filtered results, no unique DEGs were identified by NOISeq for both TNBC and ER+ (Figs. 3b and 4b). By contrast, EBSeq consistently identified the greatest number of unique DEGs for TNBC (n = 2,151; Fig. 3b) and ER+ (n = 769; Fig. 4b). Compared to EBSeq, voom, DESeq2 and edgeR identified much fewer unique DEGs. Interestingly, DEGs identified by voom were only common to EBseq and no other DGE model for TNBC, while just one overlapping DEG was also recorded for NOISeq for the ER+ contrast.

### 3.3 Comparison of two-fold filtered DGE outputs for all, and high versus lowly expressed genes

The number of DEGs detected in the all genes and highly expressed comparisons for both datasets were the same for voom (Fig. 3, Fig. 4). Thus, for both datasets no DEGs detected by voom fell below the low expression threshold. Similarly, the majority of the DEGs detected by NOISeq were those that were highly expressed with just three and two DEGs detected with low expression. DESeq2, EBSeq and EdgeR were more sensitive to detecting DEGs with low level expression and there was relatively high concordance between the genes identified by these models for both TNBC (n = 148; Fig. 3d) and ER+ (n = 100; Fig. 4d).

### 3.4 Robustness and reproducibility of DGE pipelines for GO terms

**Fig. 5:**
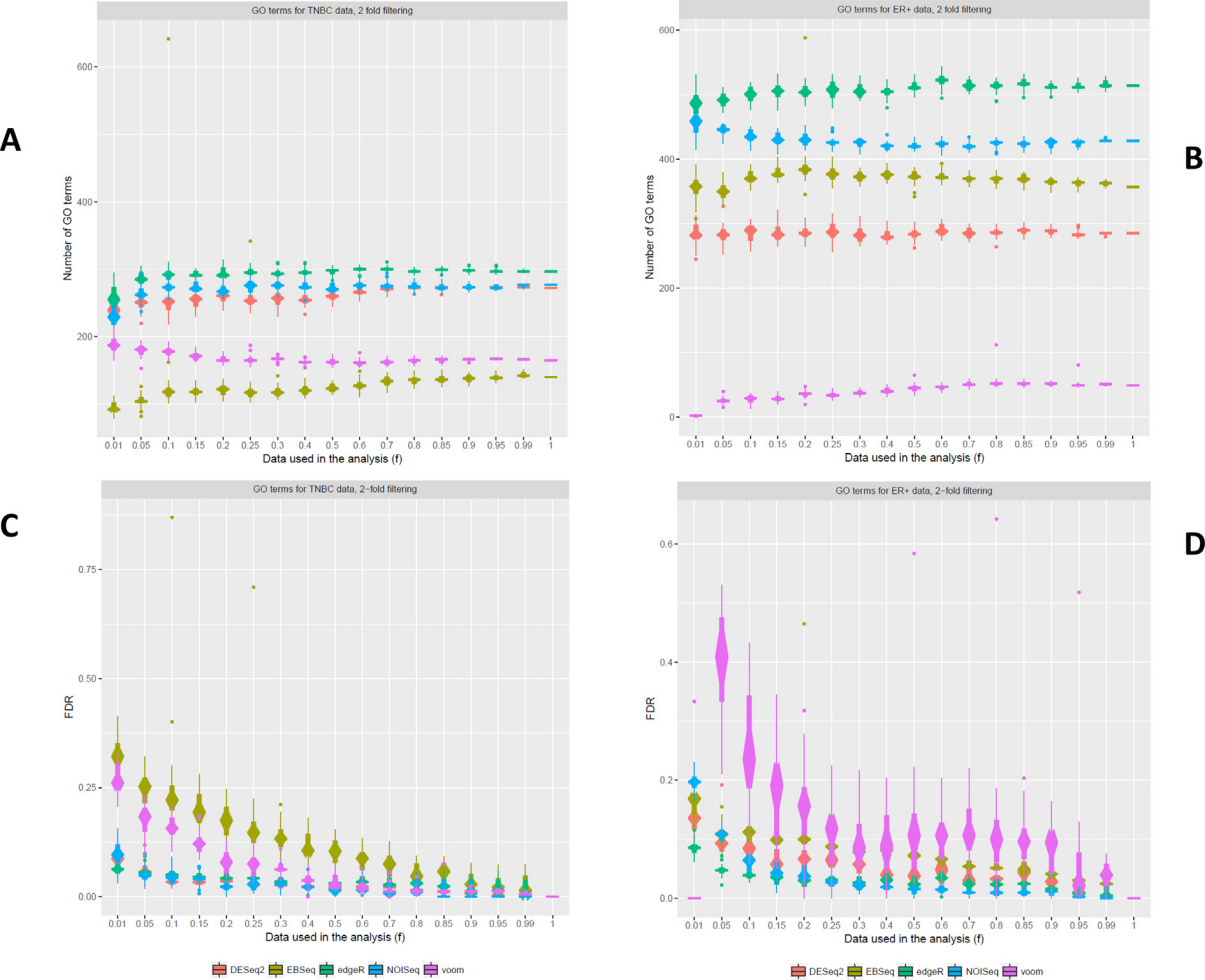
Number of GO terms detected from the TNBC and ER+ datasets – A comparison of the effect of decreased cDNA library sequencing depth on the number of GO terms detected from the TNBC and ER+ datasets (a, b) using DESeq2, edgeR, voom + limma, EBSeq and NOISeq and their associated relative FDRs (c, d).

Total number of GO terms detected differed between DGE models for both TNBC and ER+ (Fig. 5). The order of the DGE models that detected the greatest to the fewest GO terms also differed between contrasts. For TNBC, the greatest number of GO terms was detected by edgeR (~300), followed by NOIseq, DESeq2, voom and EBSeq (~140; *f* = 1; Fig. 5a). However for ER+, the greatest number of GO terms was detected by edgeR (~520), followed by NOIseq, EBSeq, DESeq2 and voom (~50; *f* = 1; Fig. 5b). The number of GO terms detected decreased with decreasing library sizes for three DGE models. However, more GO terms were detected at smaller library sizes (*f* = 0.01 – 0.4) by voom and NOISeq, respectively, in TNBC and ER+. This finding was not mirrored for voom and NOISeq in both data contrasts.

Test sensitivity for GO term detection was much lower compared to results for two-fold filtered DEGs (Fig. 1, Fig. 5). For example, when 50% of reads were analysed (*f* = 0.5) relative FDRs were approximately double the size for GO terms, compared to DEGs, for both TNBC (<0.13) and ER+ (<0.15; Fig. 5c, d). For GO outputs, NOISeq consistently had the greatest test sensitivity as indicated by the smallest relative FDRs at most library sizes; this result was observed in both contrasts. Compared to the other DGE models, EBseq and voom displayed less stability and test sensitivity as evidenced by their larger relative FDRs, confidence intervals and number of outliers. This was particularly evident in ER+ for voom (see Fig. 5d).

### 3.5 Concordance of DGE models for GO terms

**Fig. 6:**
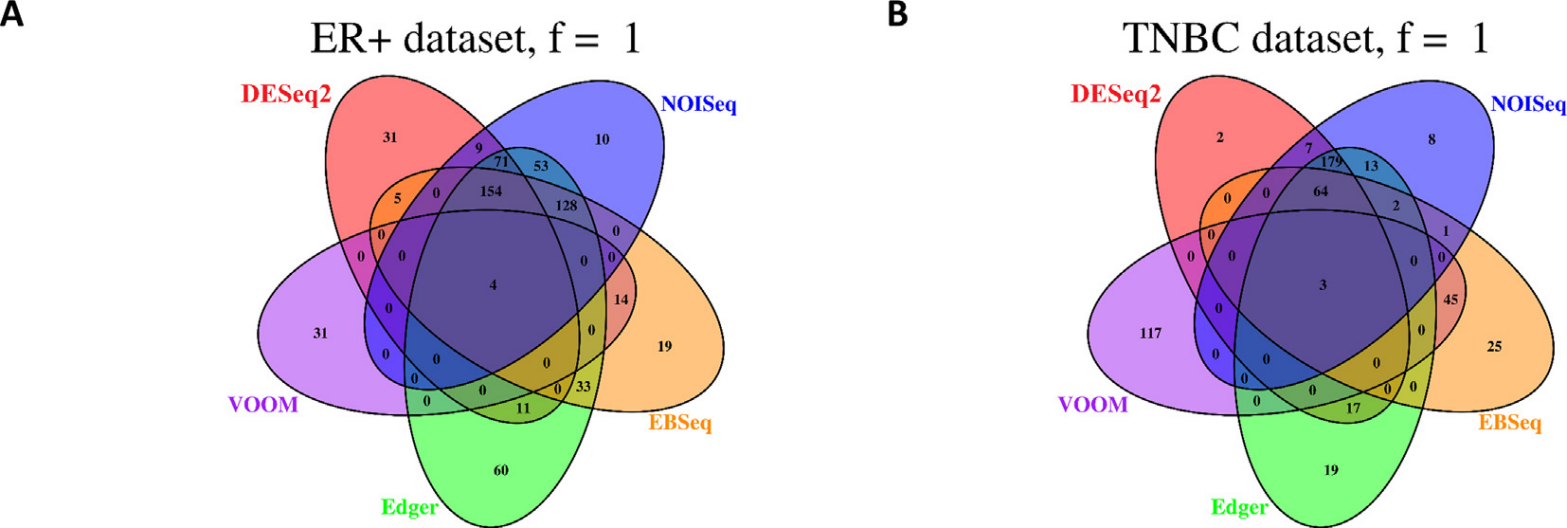
a, b. Overlap of GO terms detected from the TNBC and ER+ datasets using DESeq2, edgeR, voom + limma, EBSeq and NOISeq.

There was little concordance in GO terms detected by all DGE models from TNBC (n = 3) and ER+ (n = 4; Fig. 6). Once voom was excluded, a relatively high number of GO terms were consistently identified between DESeq2, edgeR, EBSeq and NOISeq for TNBC (n = 64). Overall, the greatest concordance was observed between DESeq2, edgeR and NOISeq; their results had the highest number of overlapping GO terms (*f* = 1; n = 179). For ER+ the greatest concordance was observed between DESeq2, edgeR, EBSeq and NOISeq (n = 154). It was interesting to note that voom consistently identified the most unique GO terms for both contrasts. With the exception of the GO terms common to all software, voom GO terms were only common with EBSeq for both contrasts, mirroring findings for DEGs. Results indicate that the majority of GO terms identified are method-specific.

### 3.6 Robustness and reproducibility of DGE pipelines comparing relative FDRs

Comparison of results for DEG detection between full and subset (5 + 5) datasets revealed different trends in DGE model stability and robustness (see Fig. 1 vs S1; Fig. 2 vs S2). Relative FDRs were much larger for subset results compared to full datasets, indicating lower test sensitivity. Test sensitivity differed between filtering regimes for the full datasets but not for the subsets. DGE models exhibited a lack of robustness for subsets as test sensitivity patterns differed between contrasts. Patterns of relative DGE model robustness were found to be dataset-dependent for reduced sample sizes. By contrast, when sample sizes were sufficiently large, results were dataset-agnostic and reliable for accurately assessing DGE model robustness to library and sample size perturbations. The figures clearly demonstrate that the robustness and reproducibility analysis outcome tends to be more dataset-dependent with sample size reduction.

**Fig. 7:**
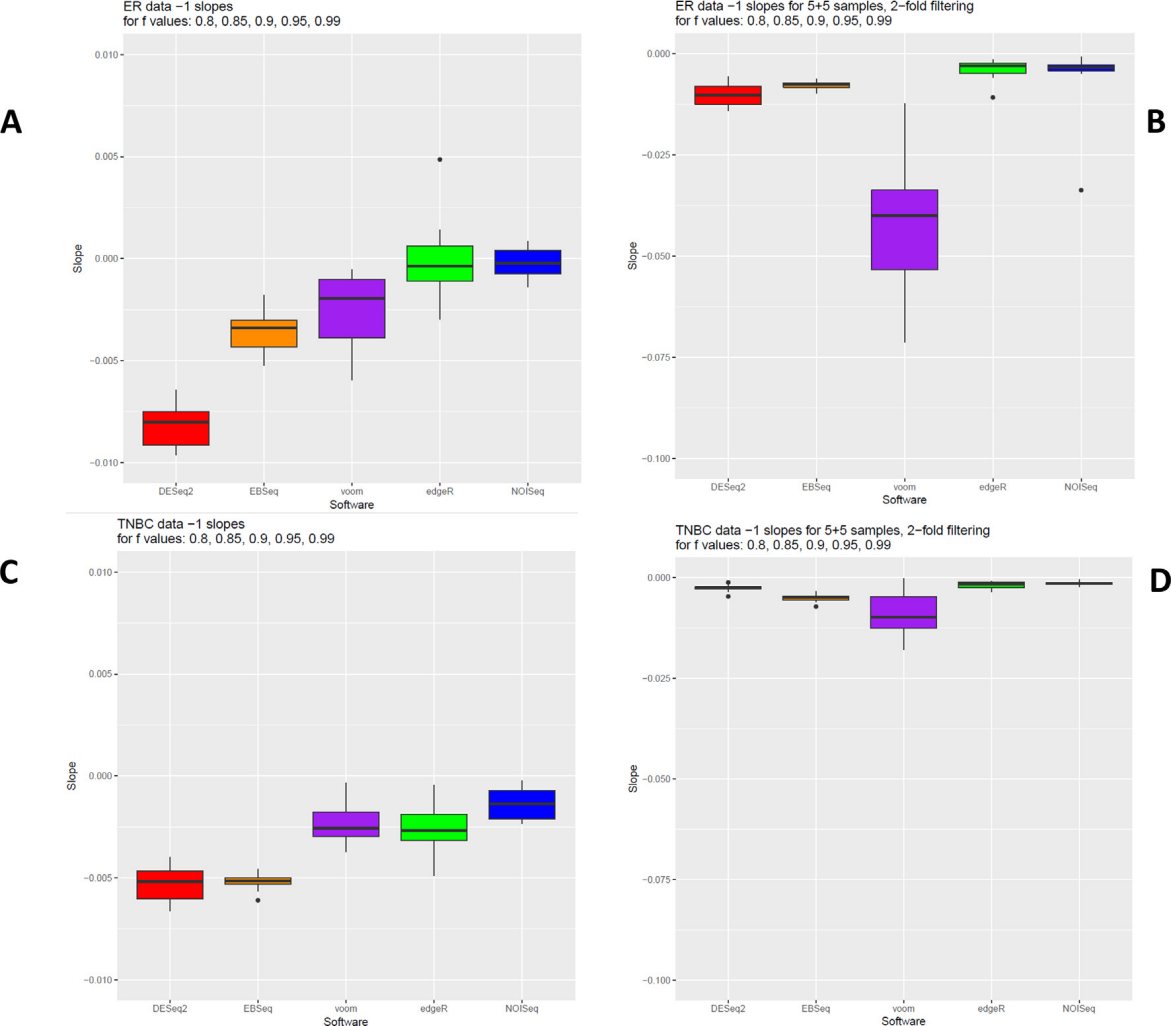
Slopes of the regression lines of the relative FDRs – A comparison of the slopes of the regression lines of the relative FDRs across differing library sizes (*f* = 0.8, 0.85, 0.9, 0.95, 0.99) obtained for analysis with DESeq2, edgeR, voom + limma, EBSeq and NOISeq for the large (All-1) and subset (5 + 5–1) comparative datasets in the ER+ (a, b) and TNBC (c, d) contrasts.

Slopes of the regression lines of relative FDRs across library sizes (*f* = 0.8 – 1) differed significantly between DGE models for large and subset comparisons for both TNBC and ER+ (*P*-values < 0.001; see Table 1, Table 2, S4, Fig. 7). When datasets were large, approximately the same pattern in slopes was detected between DGE models for both TNBC and ER+. NOISeq had a mean slope close to zero for both TNBC (−0.00133) and ER+ (−0.00017). Thus, NOISeq was the least impacted by library size reduction and hence the most robust DGE model. Conversely, DESeq2 had the largest negative slope in both TNBC (−0.00523) and ER+ (−0.00846) indicating it was the least robust model. Results for ER+ indicated that edgeR performed almost as well as NOISeq, followed by voom and EBSeq. By contrast, results for TNBC revealed that edgeR and voom performed equally well, as did EBseq with DESeq2.

**Table 1:** Results of the Friedman test to compare the slopes of the regression lines of relative FDRs between DGE models for analysis with large (All-1) and subset (5 + 5–1) comparative datasets in the TNBC and ER+ contrasts. *P*-values are two-sided according to the Student's *t*-distribution.

Dataset	Contrast	Chi-squared Test statistic	Degrees of Freedom	*P*-value
Large	TNBC	34.545	4	5.76E-07
Subset	TNBC	32.218	4	1.73E-06
Large	ER+	35.782	4	3.21E-07
Subset	ER+	37.018	4	1.79E-07

**Table 2:** Results of the post-hoc analysis with the Conover test for the pairwise comparisons of mean ranks between DGE models for large and subset comparative datasets.

Large Datasets						Subset Datasets				
**i) TNBC**		DESeq2	EBSeq	voom	edgeR		DESeq2	EBSeq	voom	edgeR
	EBSeq	NS	–	–	–	EBSeq	****	–	–	–
	voom	****	****	–	–	voom	****	NS	–	–
	edgeR	****	****	NS	–	edgeR	***	****	****	–
	NOISeq	****	****	NS	*	NOISeq	****	****	****	NS

**ii) ER+**		DESeq2	EBSeq	voom	edgeR		DESeq2	EBSeq	voom	edgeR
	EBSeq	****	–	–	–	EBSeq	***	–	–	–
	voom	****	NS	–	–	voom	****	****	–	–
	edgeR	****	****	****	–	edgeR	****	****	****	–
	NOISeq	****	****	****	NS	NOISeq	****	****	****	NS

Two-sided *P*-values *<0.05; **<0.01; ***<0.005; ****<0.001.

When datasets were smaller, the pattern in slopes detected between DGE models was not conserved between TNBC and ER+. Voom had the largest negative slope for both TNBC (−0.00895) and ER+ (−0.04083) and relatively large confidence intervals indicating it was the least robust DGE model with unstable outputs. By contrast the other DGE models all had much smaller confidence intervals suggestive of more stable outputs. For TNBC, DGE models in descending order of robustness were NOISeq (−0.00142), edgeR (−0.00192), DESeq2 (−0.00261) and EBSeq (−0.00511). Conversely for ER+, DGE models in descending order of robustness were edgeR (−0.00356), NOISeq (−0.00594), EBSeq (−0.00781) and DESeq2 (−0.01021). Post-hoc analyses revealed a large number of pairwise comparisons were significant for both the Conover and Nemenyi tests, confirming disparity between DGE models (Tables 3 and S2). Trends differed between contrasts for both the large and subset datasets. For example, Conover test results for the large TNBC dataset indicated that EBSeq and DESeq2 did not differ significantly, as voom did not with either edgeR or NOIseq. However, for the ER+ contrast these three pairwise comparisons were significantly different. Trends also differed between the large and the subset datasets for the same contrast. For example, for TNBC, mean ranks differed significantly between EBSeq and DESeq2 and between voom with both edgeR and NOIseq for the subset but not for the large dataset. For ER+, mean ranks differed significantly between EBSeq and voom for the subset but not for the large dataset.

## 4 Discussion

This study provides a comprehensive and in-depth comparison of five DGE pipelines for RNA-seq using a controlled quantitative approach. The DGE models tested were count-based and examined expression at the gene level. The analytical method used fixed count matrices as input, thus the complexity of read mapping uncertainty was excluded, as was depth of coverage to quantification of expression levels. Utilising the list of true positive DEGs from the full dataset as a reference for the estimation of relative FDR provided a relatively objective measure to compare a DGE model’s performance. Results were examined with power simulations using a range of library sizes from ~50 M (*f* = 1) to ~0.5 M (*f* = 0.01) reads per sample. These covered the minimum threshold library size of 20 M reads per sample required for an effective DE analysis [48]. Thus, test sensitivity (FDR) of a DGE method was evaluated by perturbing a real RNAseq dataset using sub-sampling and then comparing the list of DGEs obtained for the sub-sampled dataset to the full dataset (i.e. TPs). This definition of test sensitivity does not consider the DGEs obtained by full dataset, but not obtained by sub-sampled dataset (i.e. Miss-detected, MD). An alternative approach might have been to characterise test sensitivity as percentage change (PC), whereby PC = 100*(FPs + MDs) / TPs. This would have also allowed an estimation of the miss detection rate MDR = MDs / TPs = 0.99.

Previous studies found that filtering DGE results reduced FDR between findings from different laboratories and HTS platforms [8]. In this study, relative FDR was higher for two-fold filtered results, indicating that filtering results increased the disparity between DGE model outputs. Herein, findings were discordant across DGE methods similar to previous studies. This trend can worsen when replicate numbers are reduced or are more heterogeneous [28]. Results revealed that patterns of DGE model robustness were data-dependent at lower but not at larger samples sizes. Thus, comparisons of DGE model robustness were only reliable at larger library sizes, allowing conclusions to be drawn. Amongst the DGE models tested, NOISeq was the most robust, followed by edgeR, voom, EBSeq and DESeq2. NOISeq outperformed the other pipelines under differing filtering regimes and at most library sizes. However, reducing sample size notably reduced the number of DEGs detected by NOISeq and voom compared to the other pipelines and relative FDR was slightly elevated. Greatest concordance was observed between either EBSeq and voom, or DESeq2, edgeR, EBSeq and NOISeq. Both EBSeq and voom implement an empirical Bayesian approach for identifying DEGs, although the statistical distributions they use to model count data differs (NB vs Log-Normal). EBSeq identified large numbers of unique DEGs compared to the other pipelines, which identified few and NOISeq hardly any.

Observed differences in DGE method performance was more than likely in part due to the fact that pipelines implemented variable normalisation methods. NOISeq implemented using upper quartile normalisation outperformed the other methods. Conversely, Assefa *et al.* found that most normalisation methods for DE analysis performed equally well, with the exception of quantile normalization [28]. In another study, Li *et al.* found no difference between TMM, DESeq2 and Raw Count normalisations [49]. Robustness probably differed between DGE pipelines due to how software handle filtering out input data prior to analysis and its impact on FDR. Filtering low-abundance data has been considered necessary because supposedly these data provide little evidence for differential expression and may interfere with statistical approximations [11]. edgeR filters out genes with very low counts across all libraries [11]. Similarly, DESeq2 applies independent filtering of low-abundance genes prior to calculating FDR as its default approach [18]. Voom filters genes with less than ten reads across all samples and those that fail to achieve a Counts Per Million (CPM) > 1 in libraries [9]. NOISeq filters out low count features using CPM, proportion test or Wilcoxon test [16]. Unlike other methods, NOISeq takes into account the experimental design and applies the filtering criterion to remove those features that fall below the threshold from every experimental condition in the dataset. NOISeq also has a batch effect correction feature. EBseq does not filter input data [13].

Nevertheless, the greatest impact on DGE results and FDR will be linked to the model assumptions for testing DEGs, including dispersion factors for characterising mean–variance relationships. It was interesting to note that the non-parametric model NOISeq outperformed all other parametric models. Parametric models such as edgeR and DESeq2 assume that count data distributions follow a proposed distribution, usually NB. This approach tries to account for the variance in gene expression across replicates being larger than mean expression values due to over-dispersion. The alternative non-parametric DGE analytical approach of NOISeq models data noise from the samples themselves and creates a reference distribution for testing whether count numbers between two conditions represent true differential expression or noise [16]. NOISeq provided results with lower relative FDR compared to parametric approaches. Thus, modelling count data using a statistical distribution provided a less accurate representation of ‘real’ data distributions. NOISeq consistently outperformed other pipelines at different library sizes. This was not surprising as NOISeq was specifically designed to be robust to sequencing depth alterations [16]. It has been suggested that non-parametric DGE methods, such as NOISeq require a higher replicate number to perform equally well as other models [27]. Certainly, NOISeq performed less well for the subset data, particularly at lower library sizes, but in general it outperformed the other methods. Findings drawn from this study are considered against sample size; larger sample sizes may obviously draw alternate reflective conclusions. Nevertheless, results from the online tool of Assefa *et al.* provided further validation for our findings that NOISeq outperformed all other software [28]. For both mRNA and lncRNA, FDR with NOISeq was lowest amongst the software tested using each of their three simulation studies (cancer tissues, cultured cell lines, normal tissues) with variable sample sizes. The Assefa *et al.* online tool utilises RNA-seq expression data that has been simulated using a non-parametric approach that makes presumptions on data distribution. By contrast our subsampling method to create samples with lower library sizes made no such assumptions. Results of this study indicated that DGE outputs from voom were notably different to other pipelines. Voom consistently identified the greatest number of unique GO terms for different library sizes. Hence, DEGs identified by voom were very heterogeneous compared to those identified by the other pipelines. Nevertheless, some concordance was identified between voom and EBSeq, perhaps because both software apply Bayesian analyses and therefore have similar model assumptions. Few DEGs identified by NOISeq and voom had low expression levels, while it seemed that the other models were more sensitive to these genes. Assefa *et al.* found that FDR was not controlled well by many DE pipelines but improvements in sensitivity were attained for most DE tools with increasing number of replicates [28].

Generally normalisation of count data should assist with the removal of data outliers. Failing that, some DGE pipelines have an integrated method for identifying and treating outliers when testing for DEGs. This methodological difference probably accounted for some of the observed differences in DGE pipeline robustness. For example, edgeR can implement the likelihood ratio test using a ‘robustified’ approach against potential outliers (identified from the mean-NB dispersion trend) using the function *glmLRT()*. Similarly, voom + limma can be adjusted against outliers and hypervariable genes using the robust empirical Bayes options, which allow that a minority of the variances are sampled from an alternative more diffuse prior [50]. DESeq2 flags samples as outliers for each gene if their Cook’s distance is greater than the 0.99 quantile of the *F*-distribution. Depending on the frequency of the outlier in replicates, DESeq2 either removes the gene or replaces it with imputed values [18]. NOISeq initially applies a quality control step to examine RNA “Biotype distribution”. Outliers can be identified from QC diagnostic plots of count distributions across RNA biotypes and it is suggested to remove these data points prior to analysis. EBseq does not treat outliers during DEG testing.

In precision medicine, gene signatures can assist with patient stratification for treatment decision-making. Hence, accurate DGE is very important to guide patient management. Most clinically validated prognostic panels are using targeted approaches with RT-qPCR. Examples include panels for breast (MammaPrint, Oncotype DX, Prosigna), lung (GeneDx), prostate (Prolaris) and colon (ColoPrint). However, clinics are switching to whole-transcriptome sequencing in new RNA-seq assays. It isn't yet clear what DGE methods should be implemented in diagnostics to determine clinically relevant gene signatures. The analytical procedure implemented herein provided a real-world test of DGE pipelines for RNA-seq including a test of a model's sensitivity to expression levels. This framework should assist with benchmarking future developments for improving software and protocols for DGE and the standardisation of RNA-seq. Results identified reliable workflows at different library sizes and for genes of variable expression levels, information important for guiding DGE method selection for molecular diagnostics. Knowledge of software performance is informative for determining the most appropriate DGE model to apply to obtain results with the lowest FDR. This is useful in particular scenarios, such as small library or sample sizes that can impact upon molecular detection (e.g. low abundance genes, long non-coding RNAs) [28], [51]. This study focussed on alignment count-based DGE models that provide results at the gene level, alternative approaches available include assembly-based techniques that perform DGE on alignment-free quantifications [12], [19] and ML [52]. Both alignment-free and ML approaches can provide high-quality predictions [52], [53]. Indeed ML methods such as InfoGain feature selection and Logistic Regression classification are powerful and robust for DEG prediction [52]. However, oftentimes ML results consist of novel DEGs (70%) including a proportion of true positives (60%) [52]. Such findings are perhaps more useful in an exploratory context for biomarker development, rather than molecular diagnostics that require consistency. Also compared to assembly-based approaches, alignment count-based methods are more computationally efficient [53]. Thus, quicker turnaround times for molecular diagnostics could be achieved with alignment compared to other DGE approaches. Future studies should compare different approaches to determine which would be the most reliable method for molecular diagnostics to guide patient management.

## Declaration of Competing Interest

Professor Richard Kennedy receives payment as the medical director for Almac Diagnostic Services, M.S.T has recently received honoraria for advisory work in relation to the following companies: Incyte, MindPeak, QuanPathDerivatives and MSD. He is part of academia-industry consortia supported by the UK government (Innovate UK). These are all unrelated to this work. The other authors declare that they have no known competing financial interests or personal relationships that could have appeared to influence the work reported in this paper.
