# Supplementary material for: Robustness of differential gene expression analysis of RNA-seq
Source: Comput Struct Biotechnol J. 2021 May 26;19:3470–81. doi: 10.1016/j.csbj.2021.05.040 (PMC8214188; doi:10.1016/j.csbj.2021.05.040)
Supplement: Supplementary data 1 [file mmc1.docx]

**Robustness of differential gene expression analysis of RNA-seq**

Stupnikov A^1#^, McInerney CE^2^#, Savage K^2^, McIntosh SA^2^, Emmert-Streib F^3^, Kennedy R^2^, Salto-Tellez M^2^, Prise KM^2^, McArt DG^2^*

**Affiliations:**

1. Department of Biological and Medical Physics, Moscow Institute of Physics and Technology, Dolgoprudny, Russian Federation

2. Patrick G Johnson Centre for Cancer Research, Queen’s University, Belfast, Northern Ireland

3. Tampere University, Tampere, Finland

# **Authors contributed equally to this manuscript**

*** Senior corresponding author:**

Dr. Darragh G. McArt

Lecturer in Translational Bioinformatics,

Bioinformatics Group, Health Sciences Building,

Patrick G Johnson Centre for Cancer Research, Queen’s University Belfast,

97 Lisburn Road, Belfast BT9 7BL, UK.

Ph: 028 9097 2629

Email: d.mcart@qub.ac.uk

**SUPPLEMENTARY MATERIALS**

**Figures**

Figure S1. Number of DEGs detected from the TNBC subset with differing filtering regimes - A comparison of the effect of decreased cDNA library sequencing depth on the number of DEGs detected after no-fold or two-fold filtering (a,b) from the TNBC subset (5+5) using DESeq2, edgeR, voom+limma, EBSeq and NOISeq and their associated relative FDRs (c,d).


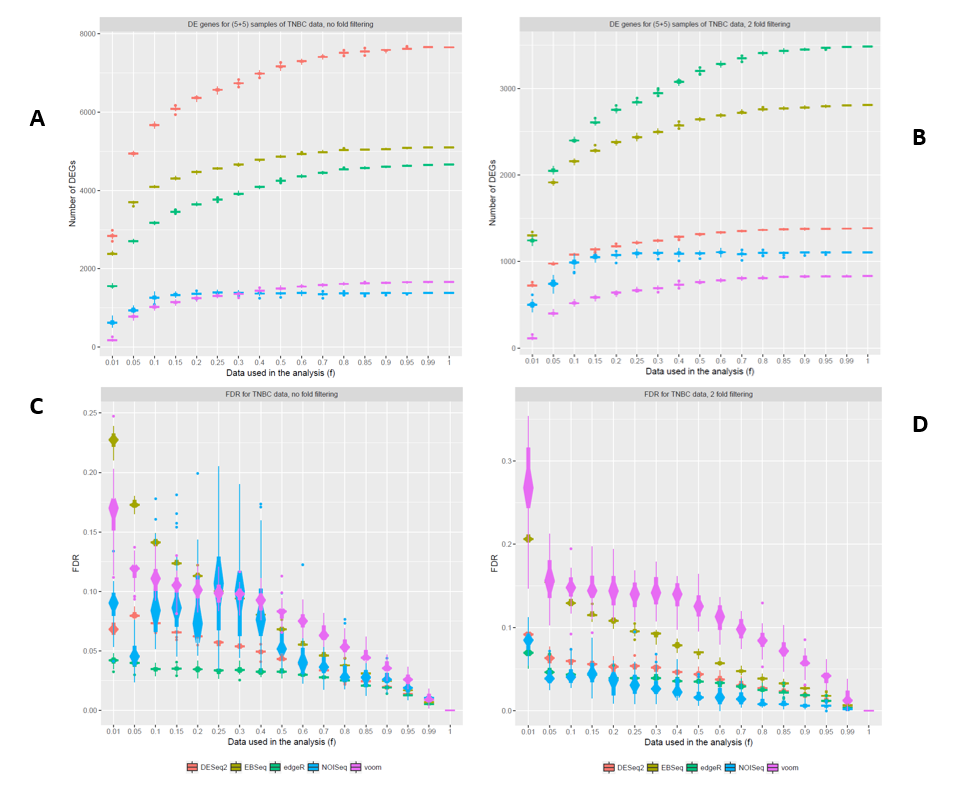


Figure S2. Number of DEGs detected from the ER+ subset with differing filtering regimes - A comparison of the effect of decreased cDNA library sequencing depth on the number of DEGs detected after no-fold or two-fold filtering (a,b) from the ER+ subset (5+5) using DESeq2, edgeR, voom+limma, EBSeq and NOISeq and their associated relative FDRs (c,d).


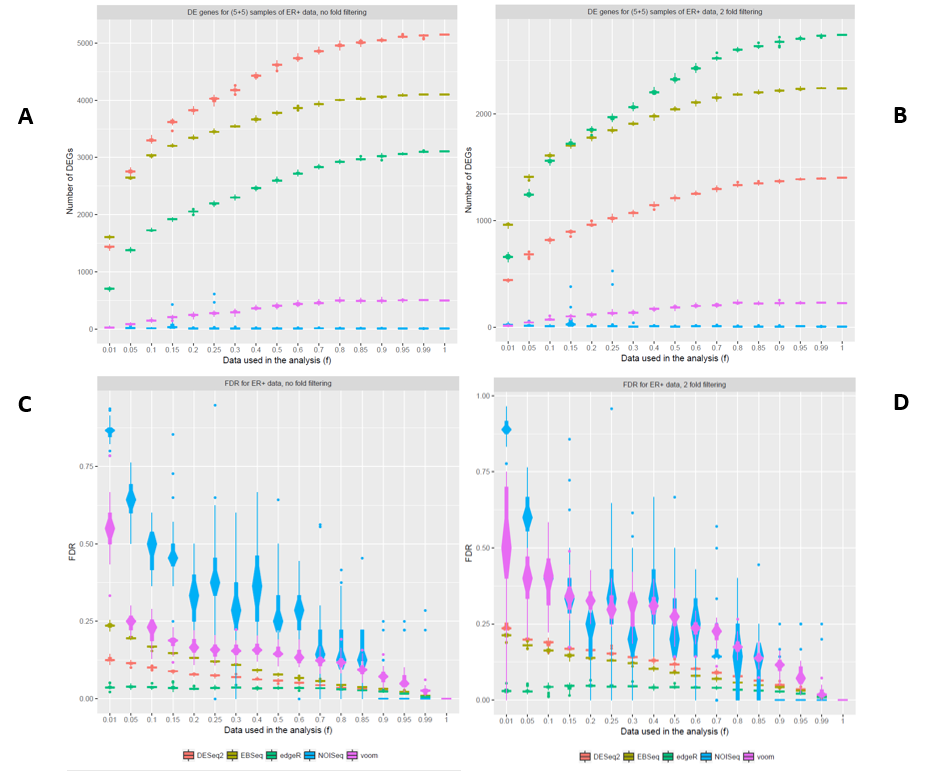


**Tables**

Table S1. Summary of the published RNA-seq samples, including GEO Accession number, that comprised the TNBC and ER+ breast cancer contrasts[see 39].

| No. | Sample Type | Sample Name | GEO Accession: |
| --- | --- | --- | --- |
| 1 | ER+ Tumor | 00-11-A037b | GSM1401676 |
| 2 | ER+ Tumor | 01-01-A061a | GSM1401677 |
| 3 | ER+ Tumor | 01-02-A056a | GSM1401678 |
| 4 | ER+ Tumor | 01-02-A325a | GSM1401679 |
| 5 | ER+ Tumor | 01-03-A207a | GSM1401680 |
| 6 | ER+ Tumor | 01-03-A373a | GSM1401681 |
| 7 | ER+ Tumor | 01-05-A181a | GSM1401682 |
| 8 | ER+ Tumor | 01-05-A359a | GSM1401683 |
| 9 | ER+ Tumor | 01-07-A281b | GSM1401684 |
| 10 | ER+ Tumor | 02-01-A013a | GSM1401685 |
| 11 | ER+ Tumor | 02-04-A362a | GSM1401686 |
| 12 | ER+ Tumor | 02-05-A203b | GSM1401687 |
| 13 | ER+ Tumor | 02-06-A431a | GSM1401688 |
| 14 | ER+ Tumor | 03-01-A341c | GSM1401689 |
| 15 | ER+ Tumor | 03-02-A013a | GSM1401690 |
| 16 | ER+ Tumor | 03-03-A069a | GSM1401691 |
| 17 | ER+ Tumor | 03-03-A117b | GSM1401692 |
| 18 | ER+ Tumor | 04-07-A414a | GSM1401693 |
| 19 | ER+ Tumor | 04-07-A432a | GSM1401694 |
| 20 | ER+ Tumor | 04-08-A204c | GSM1401695 |
| 21 | ER+ Tumor | 04-09-A066b | GSM1401696 |
| 22 | ER+ Tumor | 05-01-A256a | GSM1401697 |
| 23 | ER+ Tumor | 05-07-A186a | GSM1401698 |
| 24 | ER+ Tumor | 05-09-A159a | GSM1401699 |
| 25 | ER+ Tumor | 05-11-A240b | GSM1401700 |
| 26 | ER+ Tumor | 07-10-A110a | GSM1401701 |
| 27 | ER+ Tumor | 07-11-A250a | GSM1401702 |
| 28 | ER+ Tumor | 07-12-A271b | GSM1401703 |
| 29 | ER+ Tumor | 08-01-A301a | GSM1401704 |
| 30 | ER+ Tumor | 08-02-A355a | GSM1401705 |
| 31 | ER+ Tumor | 08-08-A142a | GSM1401706 |
| 32 | ER+ Tumor | 08-09-A192a | GSM1401707 |
| 33 | ER+ Tumor | 09-01-A214e | GSM1401708 |
| 34 | ER+ Tumor | 09-03-A118a | GSM1401709 |
| 35 | ER+ Tumor | 09-05-A135a | GSM1401710 |
| 36 | ER+ Tumor | 09-05-A149a | GSM1401711 |
| 37 | ER+ Tumor | 09-09-A106a | GSM1401712 |
| 38 | ER+ Tumor | 09-10-A057a | GSM1401713 |
| 39 | ER+ Tumor | 09-11-A112a | GSM1401714 |
| 40 | ER+ Tumor | 10-01-A051a | GSM1401715 |
| 41 | ER+ Tumor | 02-01-A055b | GSM1401716 |
| 42 | ER+ Tumor | 06-01-A009b | GSM1401717 |
| 1 | TNBC Tumor | 03-09-A268c | GSM1401718 |
| 2 | TNBC Tumor | 05-04-A096a | GSM1401719 |
| 3 | TNBC Tumor | 06-06-A277a | GSM1401720 |
| 4 | TNBC Tumor | 03-10-A451c | GSM1401721 |
| 5 | TNBC Tumor | 02-06-A195a | GSM1401722 |
| 6 | TNBC Tumor | 03-11-A307c | GSM1401723 |
| 7 | TNBC Tumor | 04-07-A416b | GSM1401724 |
| 8 | TNBC Tumor | 05-12-A078b | GSM1401725 |
| 9 | TNBC Tumor | 01-09-A215b | GSM1401726 |
| 10 | TNBC Tumor | 09-03-A053A | GSM1401727 |
| 11 | TNBC Tumor | 09-06-A120A | GSM1401728 |
| 12 | TNBC Tumor | 10-09-A101A | GSM1401729 |
| 13 | TNBC Tumor | 10-07-A138 | GSM1401730 |
| 14 | TNBC Tumor | 09-12-A158B | GSM1401731 |
| 15 | TNBC Tumor | 10-10-A177A | GSM1401732 |
| 16 | TNBC Tumor | 01-04-A048C | GSM1401733 |
| 17 | TNBC Tumor | 03-02-A158a | GSM1401734 |
| 18 | TNBC Tumor | 04-06-A164a | GSM1401735 |
| 19 | TNBC Tumor | 09-06-A089A | GSM1401736 |
| 20 | TNBC Tumor | 08-10-A297d | GSM1401737 |
| 21 | TNBC Tumor | 05-05-A201a | GSM1401738 |
| 22 | TNBC Tumor | 03-12-A386a | GSM1401739 |
| 23 | TNBC Tumor | 10-06-A120c | GSM1401740 |
| 24 | TNBC Tumor | 09-06-A139A | GSM1401741 |
| 25 | TNBC Tumor | 05-09-A223a | GSM1401742 |
| 26 | TNBC Tumor | 02-03-A078a | GSM1401743 |
| 27 | TNBC Tumor | 04-08-A183c | GSM1401744 |
| 28 | TNBC Tumor | 03-06-A283b | GSM1401745 |
| 29 | TNBC Tumor | 01-08-A054a | GSM1401746 |
| 30 | TNBC Tumor | 02-06-A263c | GSM1401747 |
| 31 | TNBC Tumor | 03-12-A222a | GSM1401748 |
| 32 | TNBC Tumor | 04-01-A168c | GSM1401749 |
| 33 | TNBC Tumor | 04-08-A020B | GSM1401750 |
| 34 | TNBC Tumor | 03-07-A523B | GSM1401751 |
| 35 | TNBC Tumor | 04-08-A101c | GSM1401752 |
| 36 | TNBC Tumor | 03-09-A087c | GSM1401753 |
| 37 | TNBC Tumor | 03-12-A154a | GSM1401754 |
| 38 | TNBC Tumor | 04-07-A389b | GSM1401755 |
| 39 | TNBC Tumor | 02-10-A335a | GSM1401756 |
| 40 | TNBC Tumor | 04-08-A019a | GSM1401757 |
| 41 | TNBC Tumor | 06-04-A286b | GSM1401758 |
| 42 | TNBC Tumor | 10-11-A138B | GSM1401759 |
| 1 | Uninvolved Breast Tissue Adjacent to ER+ Primary Tumor | 00-11-A038a | GSM1401760 |
| 2 | Uninvolved Breast Tissue Adjacent to ER+ Primary Tumor | 01-01-A062a | GSM1401761 |
| 3 | Uninvolved Breast Tissue Adjacent to ER+ Primary Tumor | 01-02-A057b | GSM1401762 |
| 4 | Uninvolved Breast Tissue Adjacent to ER+ Primary Tumor | 01-02-A326a | GSM1401763 |
| 5 | Uninvolved Breast Tissue Adjacent to ER+ Primary Tumor | 01-03-A208a | GSM1401764 |
| 6 | Uninvolved Breast Tissue Adjacent to ER+ Primary Tumor | 01-05-A183a | GSM1401765 |
| 7 | Uninvolved Breast Tissue Adjacent to ER+ Primary Tumor | 01-05-A360a | GSM1401766 |
| 8 | Uninvolved Breast Tissue Adjacent to ER+ Primary Tumor | 01-07-A283b | GSM1401767 |
| 9 | Uninvolved Breast Tissue Adjacent to ER+ Primary Tumor | 02-01-A056b | GSM1401768 |
| 10 | Uninvolved Breast Tissue Adjacent to ER+ Primary Tumor | 02-05-A204a | GSM1401769 |
| 11 | Uninvolved Breast Tissue Adjacent to ER+ Primary Tumor | 02-06-A429a | GSM1401770 |
| 12 | Uninvolved Breast Tissue Adjacent to ER+ Primary Tumor | 03-01-A342a | GSM1401771 |
| 13 | Uninvolved Breast Tissue Adjacent to ER+ Primary Tumor | 03-02-A014a | GSM1401772 |
| 14 | Uninvolved Breast Tissue Adjacent to ER+ Primary Tumor | 03-03-A070a | GSM1401773 |
| 15 | Uninvolved Breast Tissue Adjacent to ER+ Primary Tumor | 03-03-A118a | GSM1401774 |
| 16 | Uninvolved Breast Tissue Adjacent to ER+ Primary Tumor | 04-07-A434a | GSM1401775 |
| 17 | Uninvolved Breast Tissue Adjacent to ER+ Primary Tumor | 04-08-A206a | GSM1401776 |
| 18 | Uninvolved Breast Tissue Adjacent to ER+ Primary Tumor | 04-09-A067a | GSM1401777 |
| 19 | Uninvolved Breast Tissue Adjacent to ER+ Primary Tumor | 05-01-A258a | GSM1401778 |
| 20 | Uninvolved Breast Tissue Adjacent to ER+ Primary Tumor | 05-07-A187a | GSM1401779 |
| 21 | Uninvolved Breast Tissue Adjacent to ER+ Primary Tumor | 05-09-A160a | GSM1401780 |
| 22 | Uninvolved Breast Tissue Adjacent to ER+ Primary Tumor | 06-01-A010b | GSM1401781 |
| 23 | Uninvolved Breast Tissue Adjacent to ER+ Primary Tumor | 07-12-A271a | GSM1401782 |
| 24 | Uninvolved Breast Tissue Adjacent to ER+ Primary Tumor | 08-02-A356a | GSM1401783 |
| 25 | Uninvolved Breast Tissue Adjacent to ER+ Primary Tumor | 09-03-A119a | GSM1401784 |
| 26 | Uninvolved Breast Tissue Adjacent to ER+ Primary Tumor | 09-10-A058a | GSM1401785 |
| 27 | Uninvolved Breast Tissue Adjacent to ER+ Primary Tumor | 09-11-A113b | GSM1401786 |
| 28 | Uninvolved Breast Tissue Adjacent to ER+ Primary Tumor | 10-01-A052a | GSM1401787 |
| 29 | Uninvolved Breast Tissue Adjacent to ER+ Primary Tumor | 04-07-A415c | GSM1401788 |
| 30 | Uninvolved Breast Tissue Adjacent to ER+ Primary Tumor | 09-09-A107b | GSM1401789 |
| 1 | Uninvolved Breast Tissue Adjacent to TNBC Primary Tumor | 03-06-A284a | GSM1401795 |
| 2 | Uninvolved Breast Tissue Adjacent to TNBC Primary Tumor | 02-03-A079c | GSM1401796 |
| 3 | Uninvolved Breast Tissue Adjacent to TNBC Primary Tumor | 03-12-A223a | GSM1401797 |
| 4 | Uninvolved Breast Tissue Adjacent to TNBC Primary Tumor | 03-12-A387a | GSM1401798 |
| 5 | Uninvolved Breast Tissue Adjacent to TNBC Primary Tumor | 04-01-A170a | GSM1401799 |
| 6 | Uninvolved Breast Tissue Adjacent to TNBC Primary Tumor | 04-08-A102c | GSM1401800 |
| 7 | Uninvolved Breast Tissue Adjacent to TNBC Primary Tumor | 09-06-A090a | GSM1401801 |
| 8 | Uninvolved Breast Tissue Adjacent to TNBC Primary Tumor | 10-11-A139a | GSM1401802 |
| 9 | Uninvolved Breast Tissue Adjacent to TNBC Primary Tumor | 03-09-A269c | GSM1401803 |
| 10 | Uninvolved Breast Tissue Adjacent to TNBC Primary Tumor | 03-10-A452a | GSM1401804 |
| 11 | Uninvolved Breast Tissue Adjacent to TNBC Primary Tumor | 06-02-121a | GSM1401805 |
| 12 | Uninvolved Breast Tissue Adjacent to TNBC Primary Tumor | 03-12-A155a | GSM1401806 |
| 13 | Uninvolved Breast Tissue Adjacent to TNBC Primary Tumor | 04-06-A165a | GSM1401807 |
| 14 | Uninvolved Breast Tissue Adjacent to TNBC Primary Tumor | 02-06-A265a | GSM1401808 |
| 15 | Uninvolved Breast Tissue Adjacent to TNBC Primary Tumor | 04-08-A021a | GSM1401809 |
| 16 | Uninvolved Breast Tissue Adjacent to TNBC Primary Tumor | 02-10-A336b | GSM1401810 |
| 17 | Uninvolved Breast Tissue Adjacent to TNBC Primary Tumor | 03-09-A088a | GSM1401811 |
| 18 | Uninvolved Breast Tissue Adjacent to TNBC Primary Tumor | 02-04-A023a | GSM1401812 |
| 19 | Uninvolved Breast Tissue Adjacent to TNBC Primary Tumor | 01-12-A229c | GSM1401813 |
| 20 | Uninvolved Breast Tissue Adjacent to TNBC Primary Tumor | 10-09-A104a | GSM1401814 |
| 21 | Uninvolved Breast Tissue Adjacent to TNBC Primary Tumor | 01-09-A216a | GSM1401815 |

Table S2. Comparison of mean slopes of the regression lines for relative FDRs for decreasing library sizes (*f* = 0.8, 0.85, 0.9, 0.95, 0.99) for each of the DGE models for large (All-1) and subset (5+5-1) comparative datasets in the TNBC and ER+ contrasts (see Figure 8).

| Dataset | Contrast | DESeq2 | EBSeq | voom | edgeR | NOISeq |
| --- | --- | --- | --- | --- | --- | --- |
| Large | ER+ | -0.00846 | -0.00358 | -0.00263 | -0.00015 | -0.00017 |
| Large | TNBC | -0.00523 | -0.0052 | -0.00232 | -0.00255 | -0.00133 |
| Subset | ER+ | -0.01021 | -0.00781 | -0.04083 | -0.00356 | -0.00594 |
| Subset | TNBC | -0.00261 | -0.00511 | -0.00895 | -0.00192 | -0.00142 |

Table S3. Comparison of the slopes of the regression lines of the relative FDRs for large (All-1) and subset (5+5-1) comparative datasets in the TNBC and ER+ contrasts. In addition to the data removed for the subset, the same sample was removed from the larger dataset.

| i) | ER+ Large | Software | Slope All | Slope 1 | Slope 2 | Slope 3 | Slope 4 | Slope 5 | Slope 6 | Slope 7 | Slope 8 | Slope 9 | Slope 10 | Mean Slope |
| --- | --- | --- | --- | --- | --- | --- | --- | --- | --- | --- | --- | --- | --- | --- |
|  |  | DESeq2 | -0.011 | -0.008 | -0.010 | -0.009 | -0.009 | -0.009 | -0.007 | -0.006 | -0.007 | -0.008 | -0.008 | -0.009 |
|  |  | EBSeq | -0.003 | -0.003 | -0.004 | -0.003 | -0.003 | -0.002 | -0.005 | -0.003 | -0.002 | -0.005 | -0.005 | -0.004 |
|  |  | voom | -0.004 | -0.001 | -0.001 | -0.001 | -0.002 | -0.003 | -0.004 | -0.006 | -0.001 | -0.005 | -0.001 | -0.003 |
|  |  | edgeR | 0.000 | 0.000 | -0.003 | 0.000 | -0.001 | 0.001 | -0.001 | -0.001 | 0.001 | 0.005 | -0.003 | 0.000 |
|  |  | NOISeq | -0.001 | -0.001 | 0.000 | -0.001 | 0.000 | -0.001 | 0.000 | 0.001 | 0.000 | -0.001 | 0.001 | 0.000 |
|  |  |  |  |  |  |  |  |  |  |  |  |  |  |  |
| ii) | ER+ Subset | Software | Slope All | Slope 1 | Slope 2 | Slope 3 | Slope 4 | Slope 5 | Slope 6 | Slope 7 | Slope 8 | Slope 9 | Slope 10 | Mean Slope |
|  |  | DESeq2 | -0.013 | -0.010 | -0.014 | -0.008 | -0.006 | -0.010 | -0.013 | -0.008 | -0.008 | -0.010 | -0.012 | -0.010 |
|  |  | EBSeq | -0.009 | -0.007 | -0.008 | -0.008 | -0.008 | -0.007 | -0.006 | -0.008 | -0.007 | -0.009 | -0.010 | -0.008 |
|  |  | voom | -0.040 | -0.012 | -0.016 | -0.042 | -0.056 | -0.071 | -0.032 | -0.059 | -0.035 | -0.051 | -0.035 | -0.041 |
|  |  | edgeR | -0.003 | 0.001 | -0.005 | -0.002 | -0.003 | -0.006 | -0.002 | -0.005 | -0.003 | -0.001 | -0.011 | -0.004 |
|  |  | NOISeq | -0.034 | -0.002 | -0.004 | -0.002 | -0.005 | -0.001 | -0.003 | -0.003 | -0.003 | -0.003 | -0.005 | -0.006 |
|  |  |  |  |  |  |  |  |  |  |  |  |  |  |  |
| iii) | TNBC Large | Software | Slope All | Slope 1 | Slope 2 | Slope 3 | Slope 4 | Slope 5 | Slope 6 | Slope 7 | Slope 8 | Slope 9 | Slope 10 | Mean Slope |
|  |  | DESeq2 | -0.006 | -0.007 | -0.005 | -0.006 | -0.005 | -0.005 | -0.006 | -0.004 | -0.005 | -0.004 | -0.005 | -0.005 |
|  |  | EBSeq | -0.006 | -0.006 | -0.005 | -0.005 | -0.005 | -0.005 | -0.005 | -0.005 | -0.005 | -0.005 | -0.005 | -0.005 |
|  |  | voom | -0.001 | -0.003 | -0.003 | -0.003 | -0.002 | -0.003 | 0.000 | -0.002 | -0.003 | -0.001 | -0.004 | -0.002 |
|  |  | edgeR | -0.003 | -0.004 | -0.002 | -0.001 | -0.003 | -0.005 | -0.003 | -0.002 | -0.003 | 0.000 | -0.002 | -0.003 |
|  |  | NOISeq | -0.002 | 0.000 | -0.001 | -0.002 | -0.001 | -0.001 | -0.002 | -0.002 | -0.001 | 0.000 | -0.001 | -0.001 |
|  |  |  |  |  |  |  |  |  |  |  |  |  |  |  |
| iv) | TNBC Subset | Software | Slope All | Slope 1 | Slope 2 | Slope 3 | Slope 4 | Slope 5 | Slope 6 | Slope 7 | Slope 8 | Slope 9 | Slope 10 | Mean Slope |
|  |  | DESeq2 | -0.003 | -0.003 | -0.002 | -0.003 | -0.005 | -0.002 | -0.003 | -0.002 | -0.003 | -0.002 | -0.001 | -0.003 |
|  |  | EBSeq | -0.006 | -0.003 | -0.004 | -0.006 | -0.007 | -0.005 | -0.006 | -0.005 | -0.005 | -0.005 | -0.005 | -0.005 |
|  |  | voom | -0.011 | -0.008 | -0.003 | -0.014 | 0.000 | -0.006 | -0.014 | -0.004 | -0.010 | -0.011 | -0.018 | -0.009 |
|  |  | edgeR | -0.002 | -0.001 | -0.002 | -0.003 | -0.003 | -0.002 | -0.001 | -0.001 | -0.004 | -0.001 | -0.001 | -0.002 |
|  |  | NOISeq | -0.001 | -0.002 | -0.001 | -0.001 | -0.002 | -0.002 | -0.002 | -0.001 | -0.001 | -0.001 | -0.002 | -0.001 |

Table S4. Results of the Friedman test post-hoc analysis with the Nemenyi test for pairwise comparisons of mean ranks between DGE models for large and subset comparative datasets.

| **Large Dataset** |  |  |  |  |  |  | **Subset Datasets** |  |  |  |  |
| --- | --- | --- | --- | --- | --- | --- | --- | --- | --- | --- | --- |
| **i) TNBC** |  | DESeq2 | EBSeq | voom | edgeR |  |  | DESeq2 | EBSeq | voom | edgeR |
|  | EBSeq | NS | - | - | - |  | EBSeq | NS | - | - | - |
|  | voom | *** | *** | - | - |  | voom | NS | NS | - | - |
|  | edgeR | * | * | NS | - |  | edgeR | NS | *** | *** | - |
|  | NOISeq | **** | **** | NS | NS |  | NOISeq | NS | **** | **** | NS |
|  |  |  |  |  |  |  |  |  |  |  |  |
| **ii) ER+** |  | DESeq2 | EBSeq | voom | edgeR |  |  | DESeq2 | EBSeq | voom | edgeR |
|  | EBSeq | NS | - | - | - |  | EBSeq | NS | - | - | - |
|  | voom | * | NS | - | - |  | voom | NS | * | - | - |
|  | edgeR | **** | * | NS | - |  | edgeR | ** | NS | **** | - |
|  | NOISeq | **** | * | NS | NS |  | NOISeq | * | NS | **** | NS |

Two-sided *P*-value *<0.05; **<0.01; ***<0.005; ****<0.001
